# Supplementary material for: Study on microwave ablation temperature prediction model based on grayscale ultrasound texture and machine learning
Source: PLoS One. 2024 Sep 25;19(9):e0308968. doi: 10.1371/journal.pone.0308968 (PMC11423965; doi:10.1371/journal.pone.0308968)
Supplement: S1 Fig — (PDF) [file pone.0308968.s003.pdf]

Cross-sectional photos of liver post-ablation for 15 W and 20 W power Groups

15 W power group

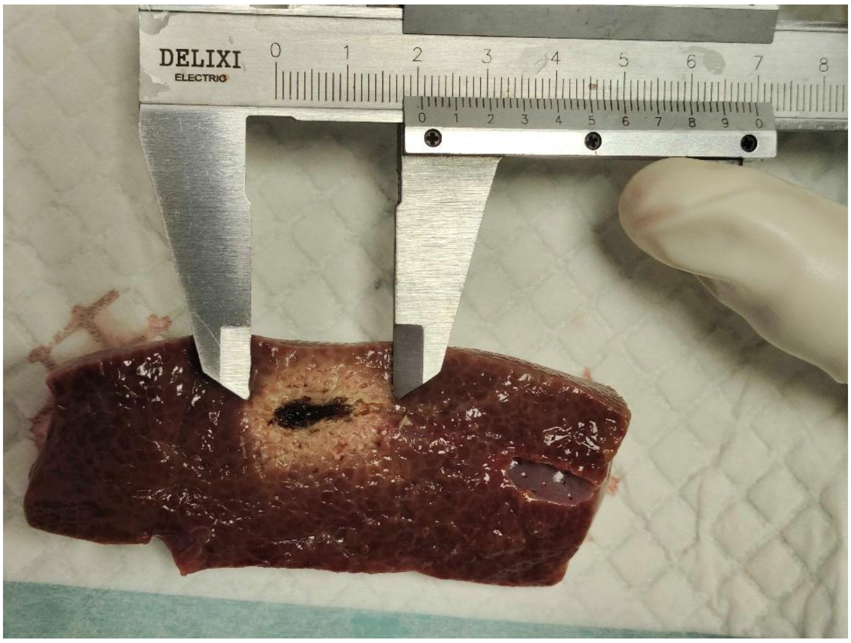

15 W-1

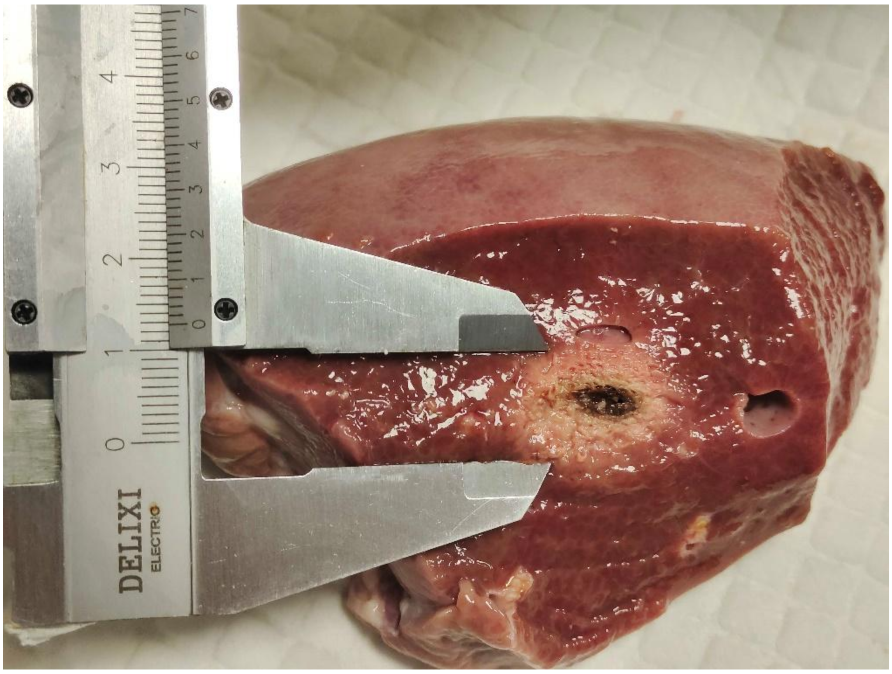

15 W-2

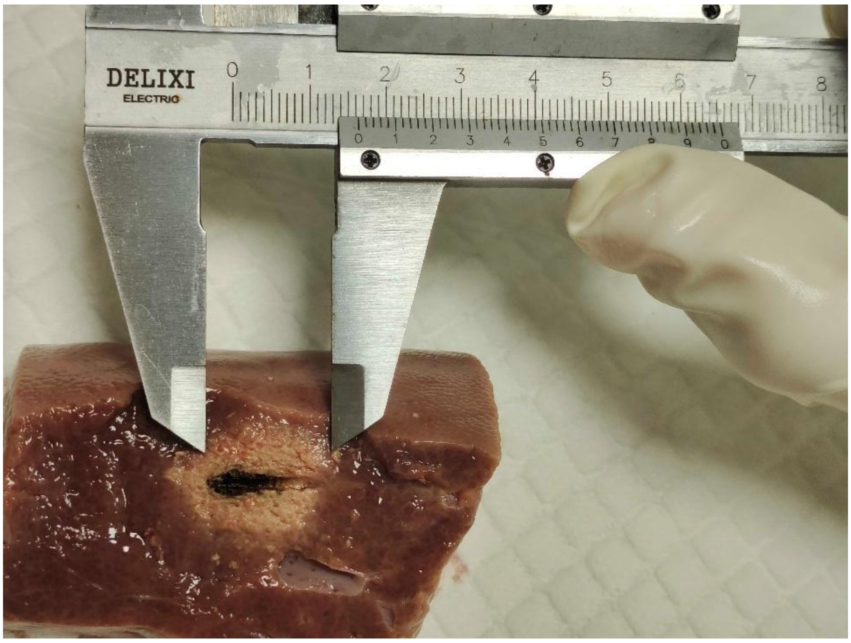

15 W-3

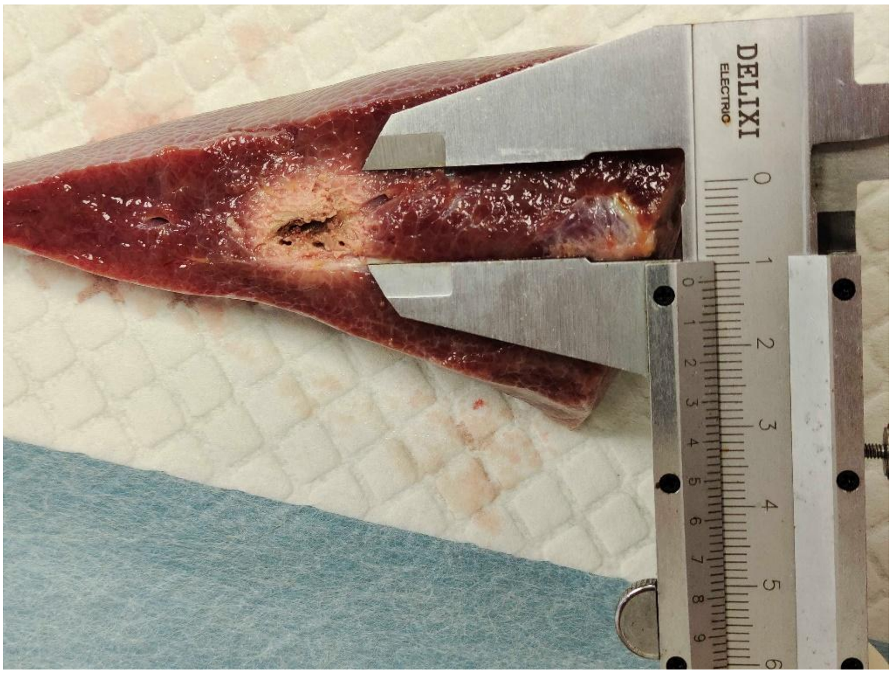

15 W-4

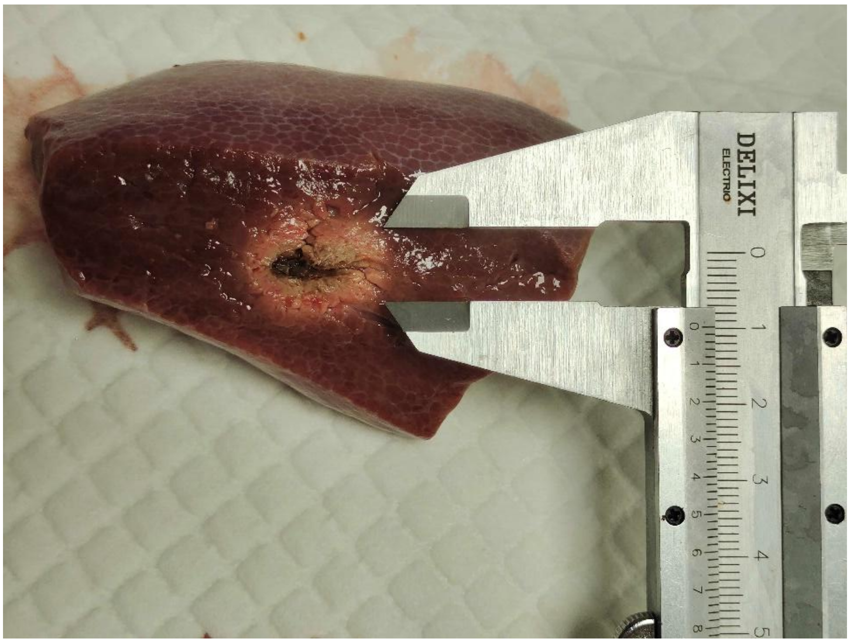

15 W-5

20 W power group

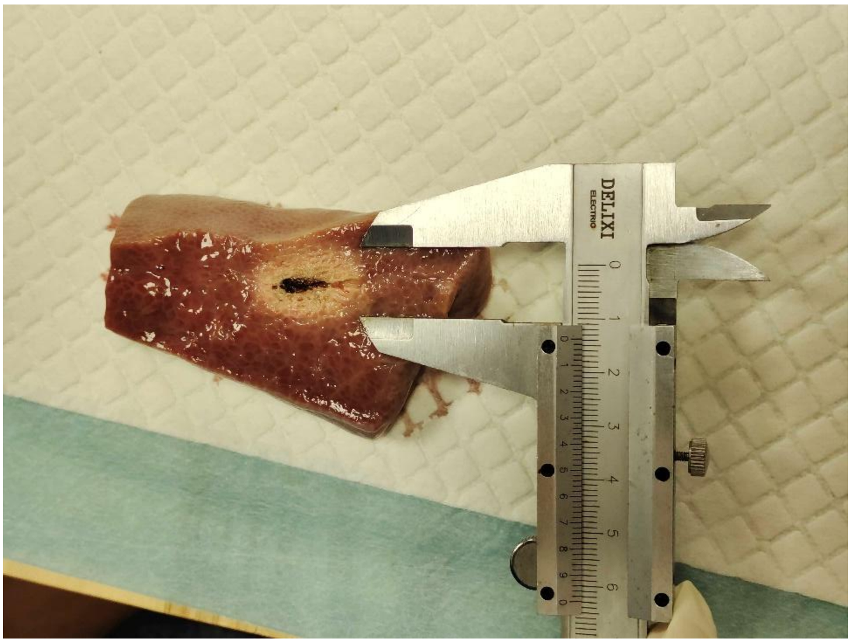

20 W-1

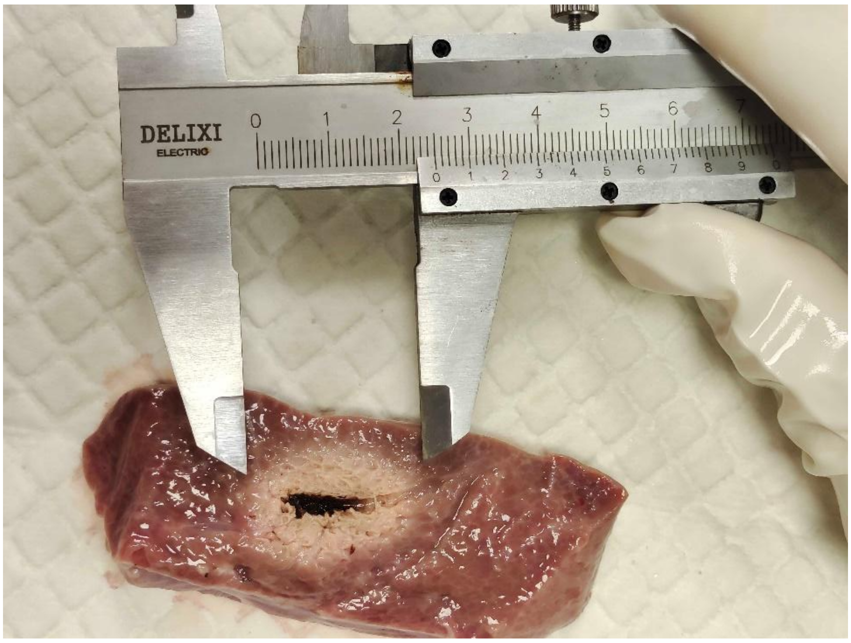

20 W-2

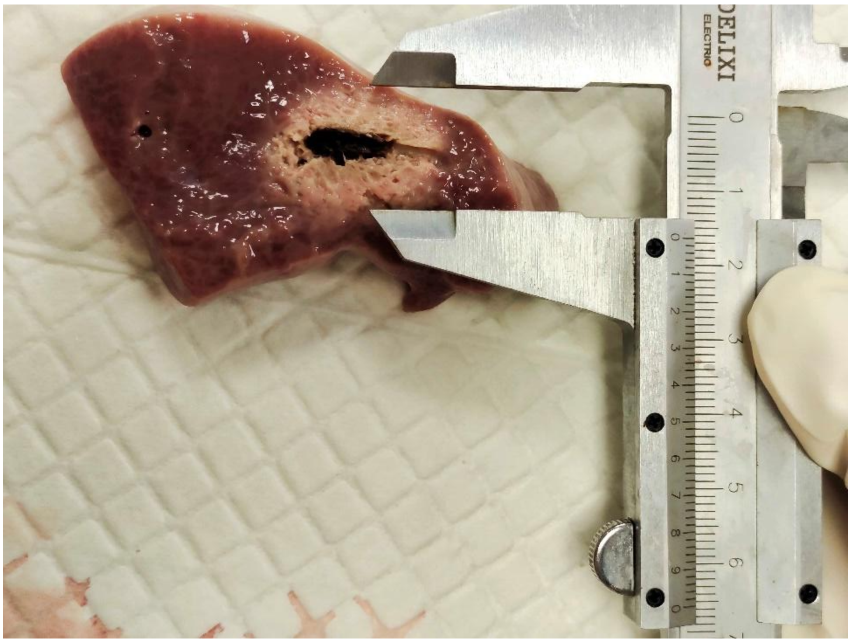

20 W-3

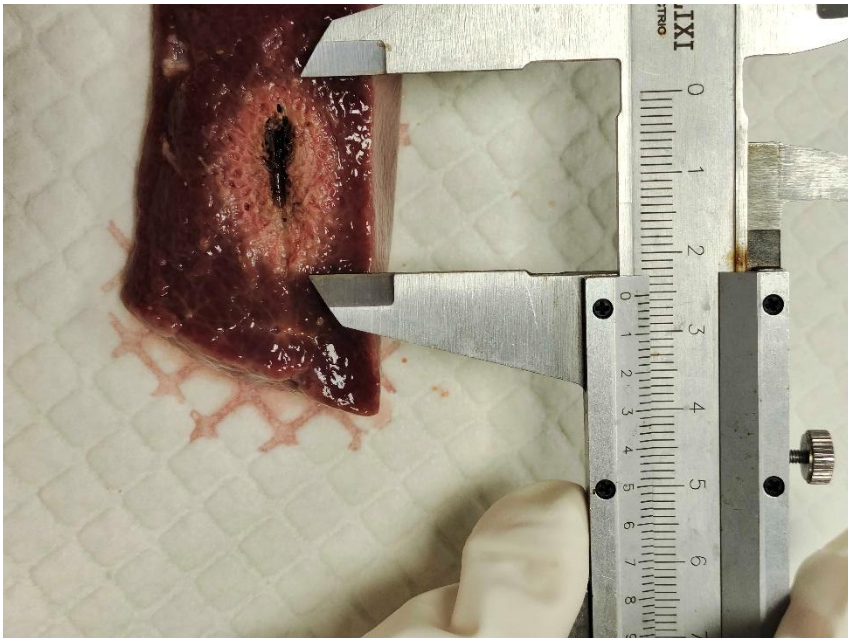

20 W-4

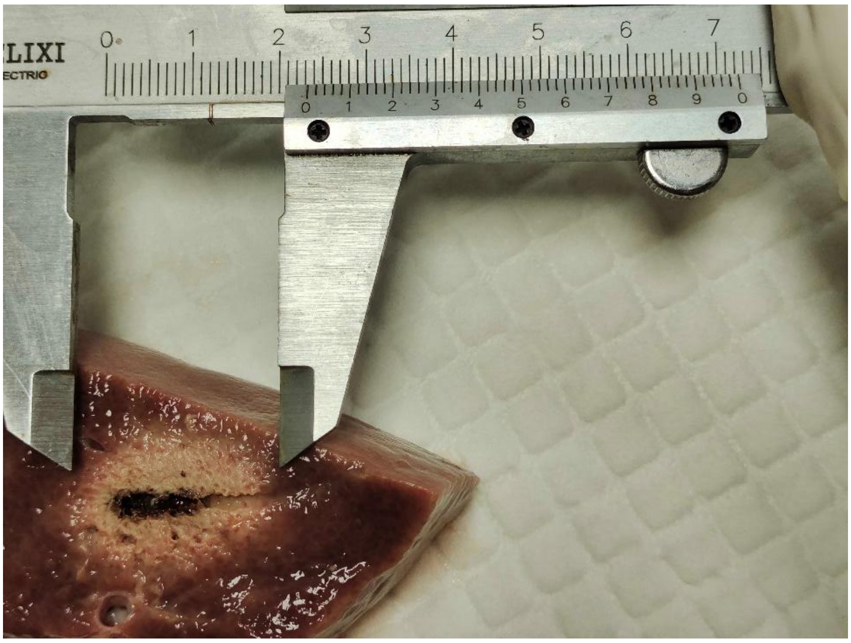

20 W-5
