# Supplementary material for: Study on microwave ablation temperature prediction model based on grayscale ultrasound texture and machine learning
Source: PLoS One. 2024 Sep 25;19(9):e0308968. doi: 10.1371/journal.pone.0308968 (PMC11423965; doi:10.1371/journal.pone.0308968)
Supplement: S2 Fig — (PDF) [file pone.0308968.s004.pdf]

Thermal maps for 15 W and 20 W power groups: machine learning predictions and 54°C threshold highlights

15 W power group

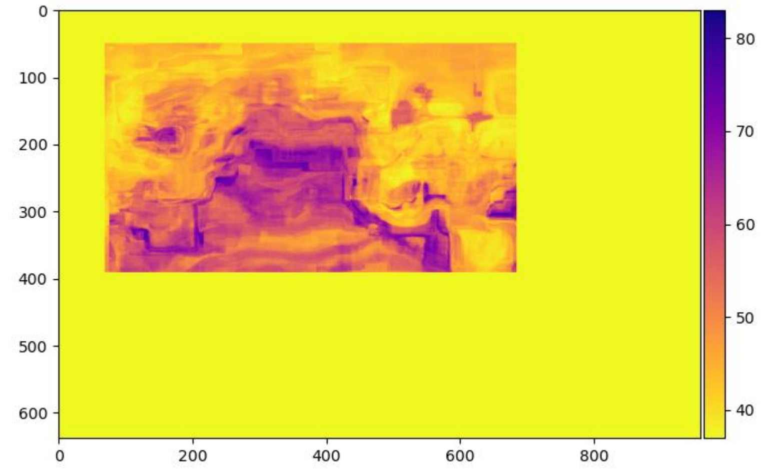

machine learning predictions： 15 W-1

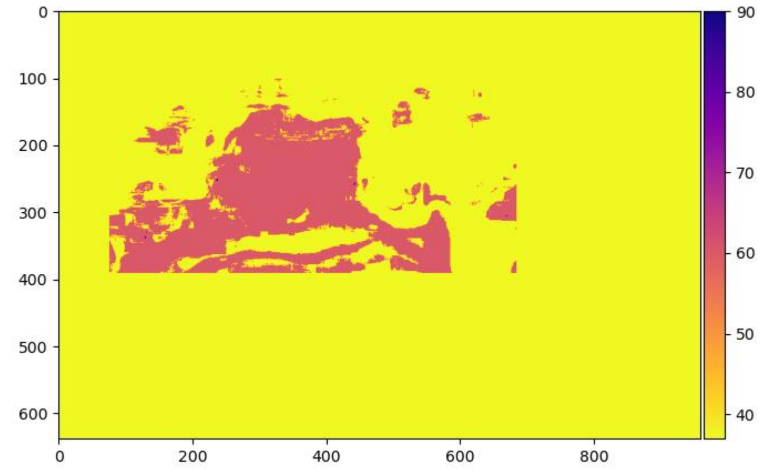

54°C threshold highlights： 15 W-1

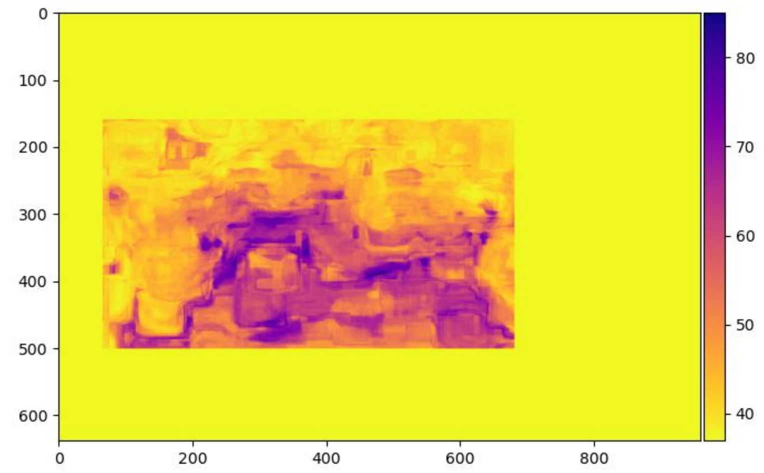

machine learning predictions：15 W-2

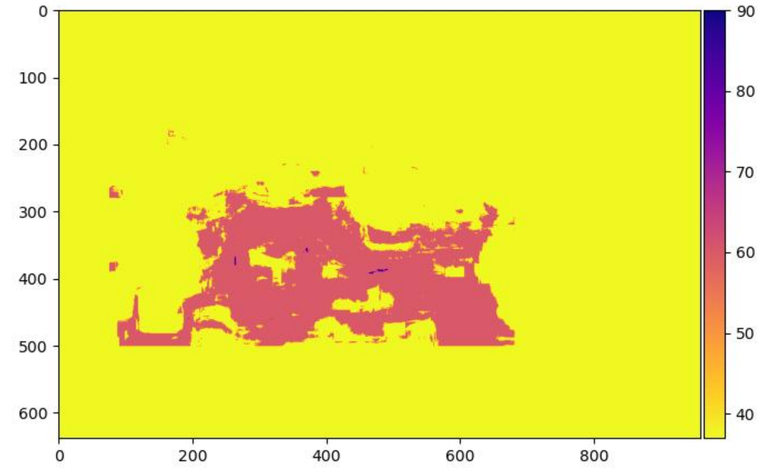

54°C threshold highlights：15 W-2

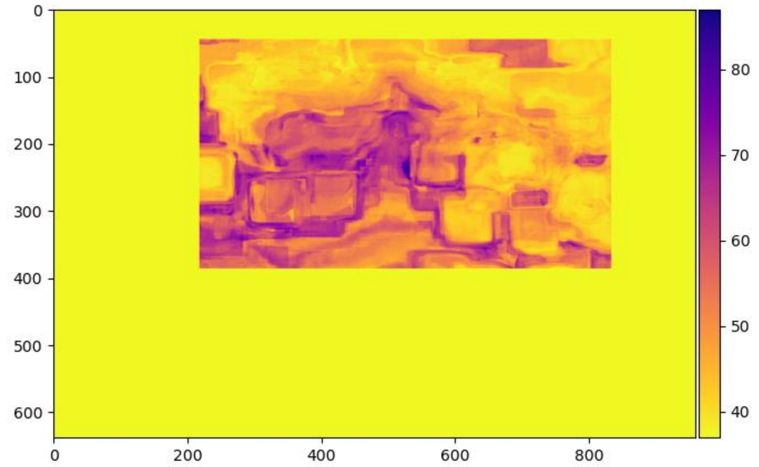

machine learning predictions：15 W-3

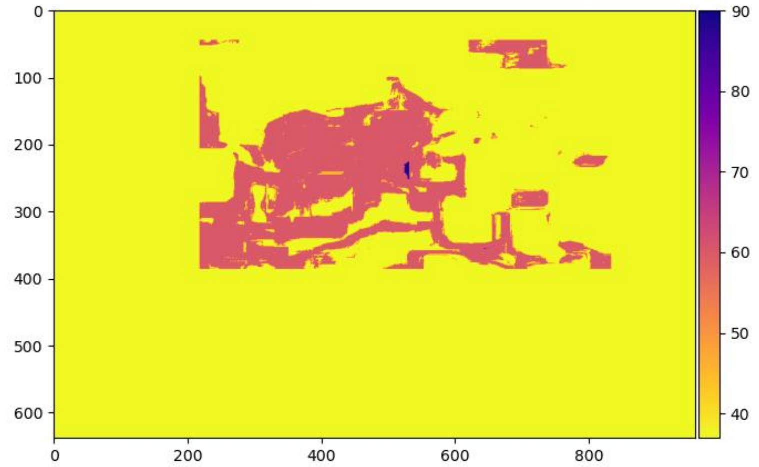

54°C threshold highlights：15 W-3

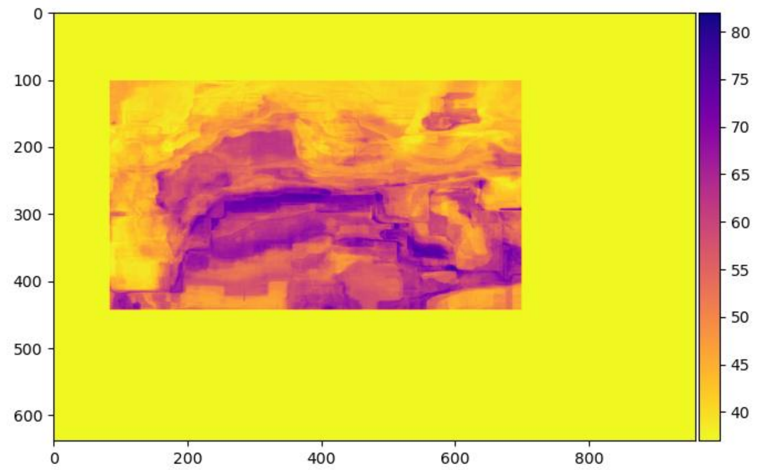

machine learning predictions： 15 W-4

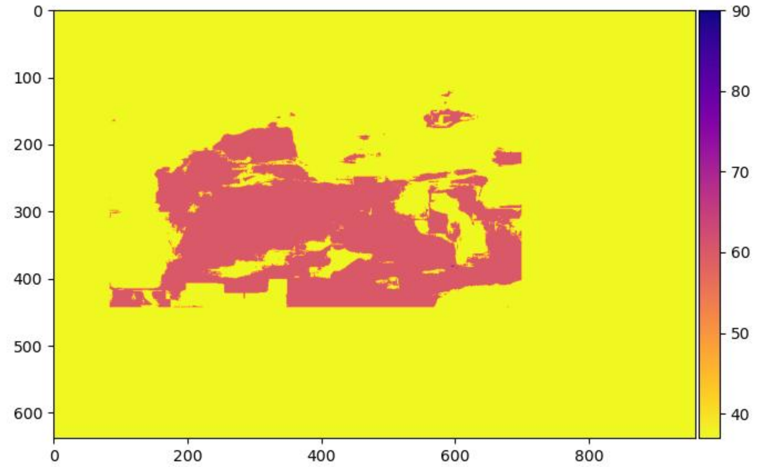

54°C threshold highlights： 15 W-4

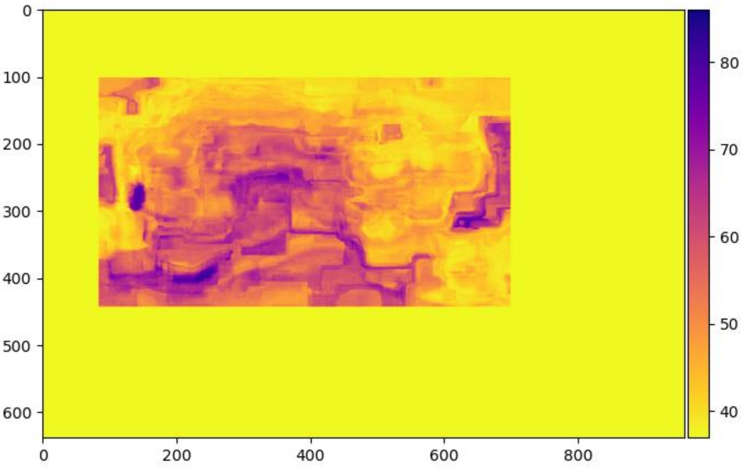

machine learning predictions： 15 W-5

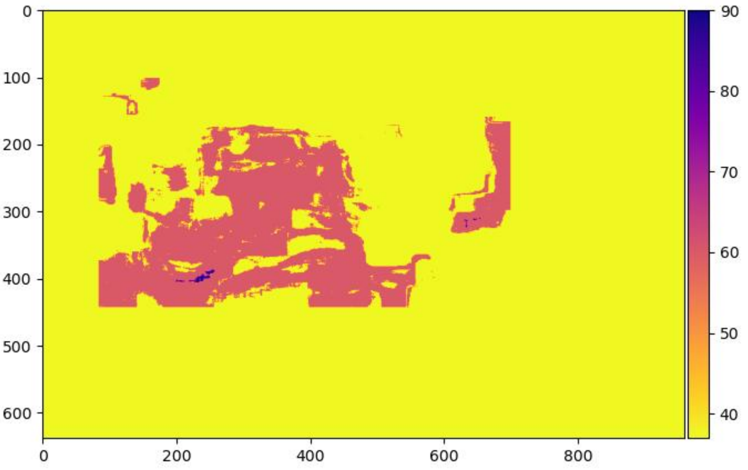

54°C threshold highlights： 15 W-5

20 W power group

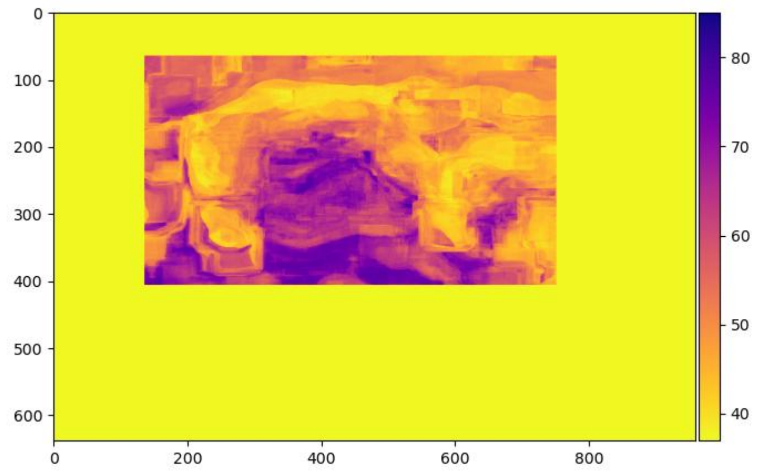

machine learning predictions：20 W-1

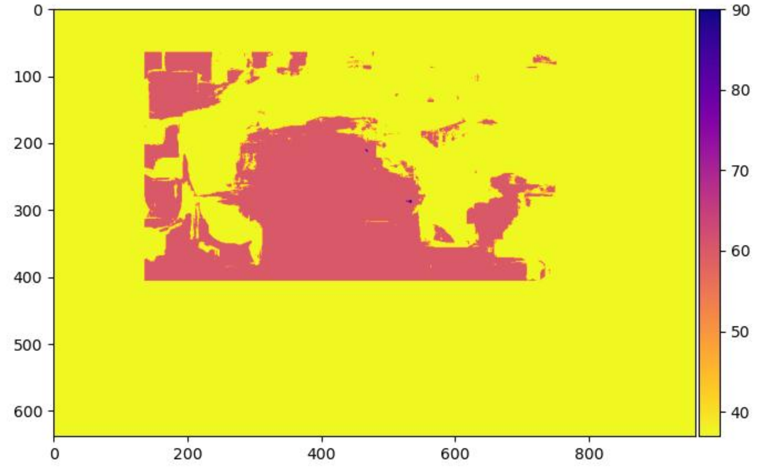

54°C threshold highlights：20 W-1

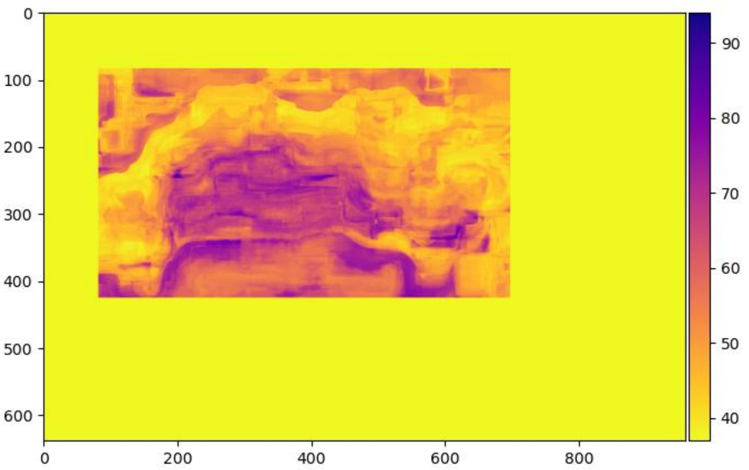

machine learning predictions： 20 W-2

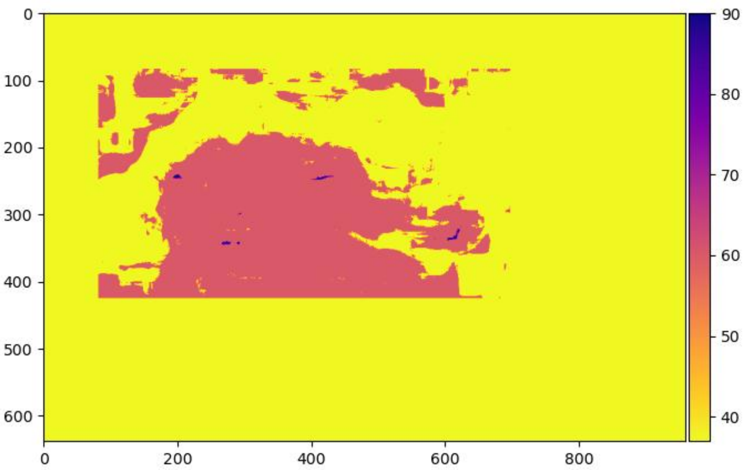

54°C threshold highlights： 20 W-2

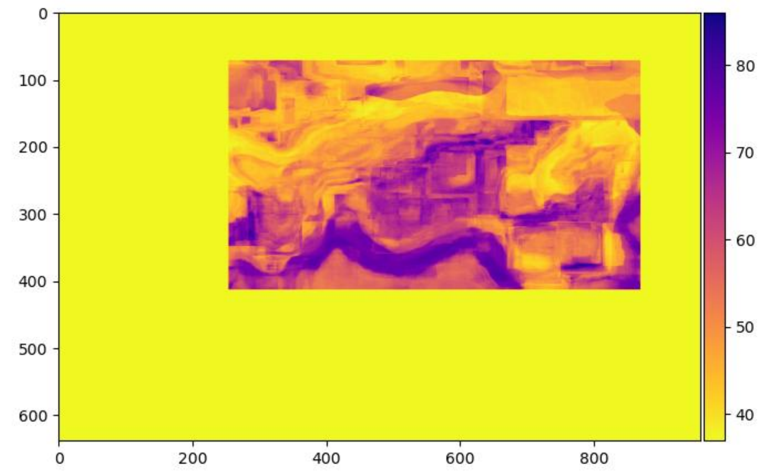

machine learning predictions：20 W-3

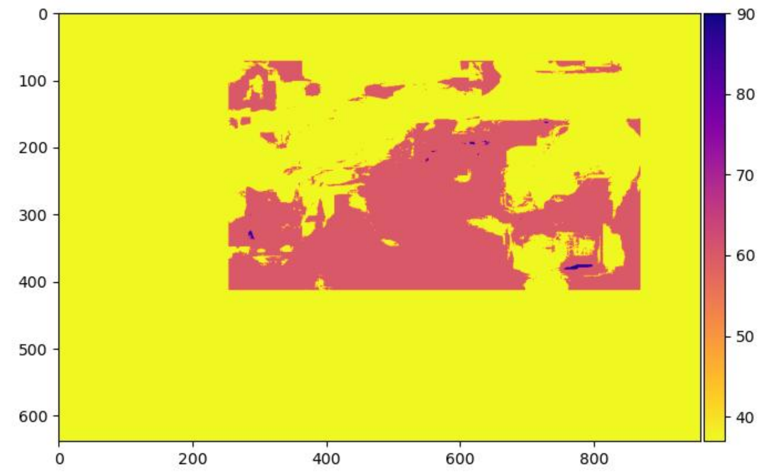

54°C threshold highlights：20 W-3

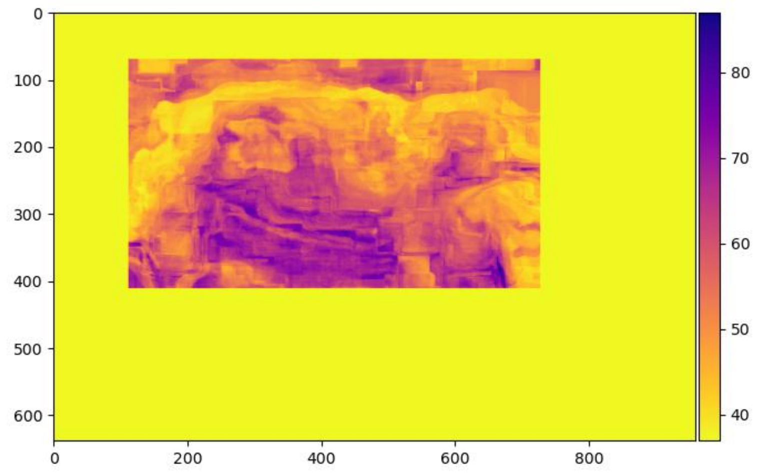

machine learning predictions： 20 W-4

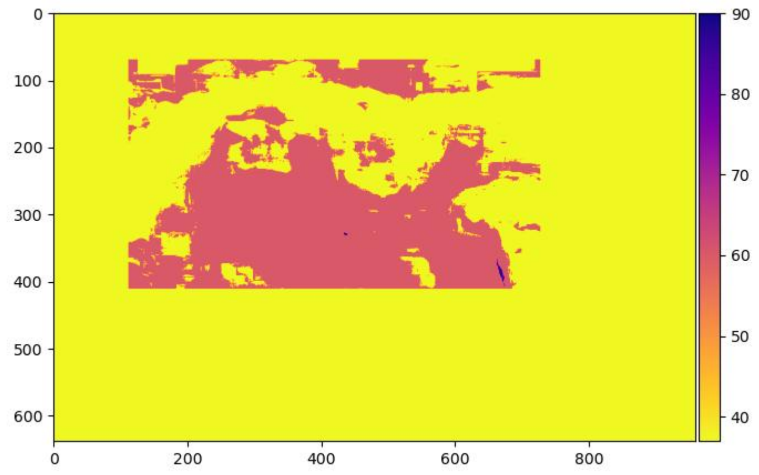

54°C threshold highlights： 20 W-4

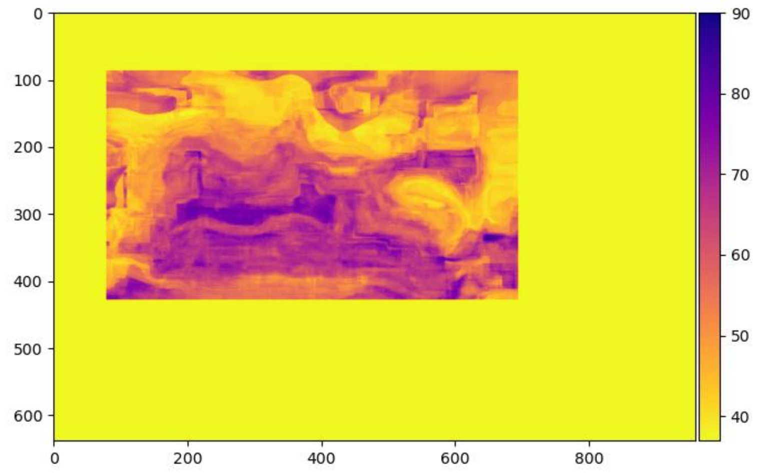

machine learning predictions： 20 W-5

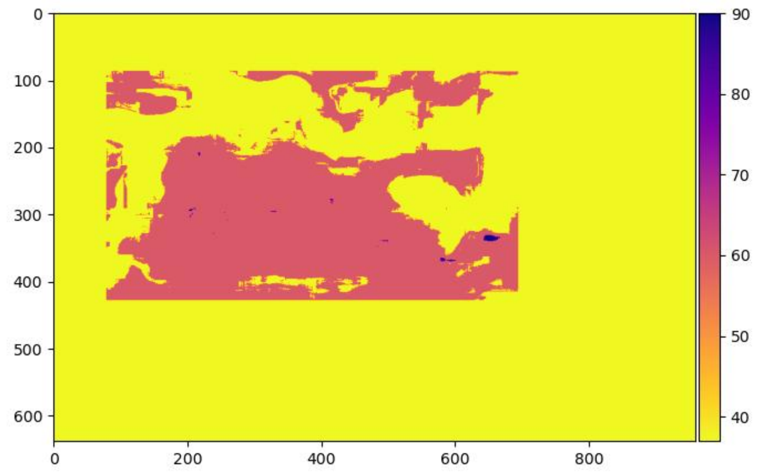

54°C threshold highlights： 20 W-5
